# Clinical and Genetic Characterization of Isolated Methylmalonic Acidemia in Malaysian Children: Identification of Two Novel *MMUT* Variants

**DOI:** 10.3390/diagnostics16050755

**Published:** 2026-03-03

**Authors:** Mardhiah Masri, Norzahidah Khalid, Noornatisha Salleh, Seok-Hian Lua, Nor Azimah Abdul Azize, Yusnita Yakob, Ernie Zuraida Ali, Vani A/P Munusamy, Lock-Hock Ngu, Jeffrey Soon-Yit Lee, Teck-Hock Toh, Anasufiza Habib

**Affiliations:** 1 Biochemistry Unit, Specialised Diagnostic Centre, Institute for Medical Research, National Institutes of Health, Shah Alam 40170, Malaysia; zahidah.k@moh.gov.my (N.K.); noornatisha@moh.gov.my (N.S.); anasufiza@moh.gov.my (A.H.); 2 Molecular Diagnostics Unit, Specialised Diagnostic Centre, Institute for Medical Research, National Institutes of Health, Shah Alam 40170, Malaysia; luaseokhian@moh.gov.my (S.-H.L.); azimahazize@moh.gov.my (N.A.A.A.); yusnita.yakob@moh.gov.my (Y.Y.); 3 Inborn Error of Metabolism and Genetic Unit, Institute for Medical Research, National Institutes of Health, Shah Alam 40170, Malaysia; ernie@moh.gov.my; 4 Biochemical Genetic Unit, Department of Pathology, Hospital Tunku Azizah, Kuala Lumpur 50300, Malaysia; vani@moh.gov.my; 5 Department of Genetics, Hospital Kuala Lumpur, Kuala Lumpur 50300, Malaysia; ngu.lockhock@moh.gov.my; 6 Department of Pediatrics, Hospital Sibu, Sibu 96000, Sarawak, Malaysia; jeffsylee@yahoo.com (J.S.-Y.L.); tohth@moh.gov.my (T.-H.T.)

**Keywords:** methylmalonic acidemia, Sanger sequencing, genetic variants, metabolic disorder, Malaysia, inborn errors of metabolism

## Abstract

**Background/Objectives**: Isolated methylmalonic acidemia (iMMA) is a rare autosomal recessive metabolic disorder caused by defects in methylmalonyl-CoA mutase (MCM) activity or in the biosynthesis of its cofactor, adenosylcobalamin. Mutations in five genes—*MMUT*, *MMAA*, *MMAB*, *MMADHC*, and *MCEE*—are known to underlie this condition. This study aimed to characterize the clinical features and molecular spectrum of iMMA in Malaysian patients of diverse ethnic backgrounds. **Material and Methods**: Patients with biochemical evidence suggestive of iMMA, including elevated propionylcarnitine (C3), increased C3/C2 ratio, and raised urine methylmalonic acid levels in the absence of hyperhomocysteinemia, were selected for genetic testing. Sanger sequencing was performed to identify pathogenic variants in the *MMUT*, *MMAA*, *MMAB*, *MMADHC*, or *MCEE* genes. **Results**: The cohort consisted predominantly of Iban patients (*n* = 5), with the remaining cases comprising one Malay and one Thai–Malay individual. Age at diagnosis ranged from Day 1 of life to 6 years. All 7 patients were confirmed to have iMMA through molecular analysis. A total of seven pathogenic or likely pathogenic variants were identified, including two novel *MMUT* variants (c.246_250delinsGA and c.1358G>C), four known *MMUT* variants (c.560C>G, c.693C>G, c.982C>T, c.1106G>A), and one known *MMAB* variant (c.644+1G>A). Clinical presentation and disease severity varied across cases, reflecting underlying genotypic heterogeneity. **Conclusions**: This study highlights the molecular diversity and clinical variability of iMMA in Malaysia. Our findings reinforce the importance of integrating metabolic screening with molecular diagnostics to identify disease-causing variants and guide patient management strategies effectively.

## 1. Introduction

MMA is a rare autosomal recessive metabolic disorder caused by defects in the conversion of methylmalonyl-CoA to succinyl-CoA, catalyzed by methylmalonyl-CoA mutase (MCM). The enzyme requires adenosylcobalamin as a cofactor, and pathogenic variants in *MMUT* or genes involved in cobalamin metabolism (*MMAA*, *MMAB*, *MMADHC*, *MCEE*) disrupt this process, leading to toxic accumulation of methylmalonic acid. When this occurs without hyperhomocysteinemia, the condition is classified as isolated MMA (iMMA) [1].

The clinical spectrum is broad. Infantile-onset cases often present with acute metabolic decompensation, encephalopathy, or sepsis-like illness, while later-onset forms may manifest with developmental delay, epilepsy, or failure to thrive. Genotypically, *MMUT*-related iMMA may present as partial (*mut*^−^) or complete (*mut*^0^) enzyme deficiency, with the latter associated with severe outcomes. Defects in *MMAA*, *MMAB*, or *MMADHC* result in cblA, cblB, or cblD-variant 2 subtypes, whereas *MCEE* variants typically cause milder disease [2,3].

The reported prevalence of MMA varies across populations, from 0.8 per 100,000 in the Asia-Pacific to 2.5 per 100,000 in Suzhou, China. In Malaysia, preliminary pilot data indicate a higher estimated prevalence of approximately 6.6 per 100,000 births [4], though the true burden remains unknown due to the absence of national universal expanded newborn screening [5,6].

Diagnosis relies on biochemical markers such as elevated C3, C3/C2 ratio, and urinary methylmalonic acid, with molecular testing required for definitive classification. Management typically includes dietary protein restriction, precursor-free amino acid formulas, and carnitine supplementation, though prognosis is strongly influenced by genotype and subtype [2].

Isolated methylmalonic acidemia (iMMA) is primarily caused by biallelic pathogenic variants in the *MMUT* gene (MIM: 609058), leading to partial (*mut*^−^) or complete (*mut*^0^) deficiency of methylmalonyl-CoA mutase (MCM, EC 5.4.99.2). The *mut*^−^ subtype typically retains partial enzymatic activity, is often B12-responsive, and is generally associated with a milder clinical phenotype. In contrast, the *mut*^0^ subtype results in complete enzymatic deficiency, is usually non–B12-responsive, and is linked to more severe outcomes, including higher risks of morbidity, mortality, and long-term complications [7]. Defects in the synthesis or transport of the MCM cofactor 5′-deoxyadenosylcobalamin (AdoCbl) can also cause iMMA, classified as cblA, cblB, or cblD-variant 2, due to variants in *MMAA* (MIM: 607481), *MMAB* (MIM: 607568), or *MMADHC* (MIM: 611935), respectively. Rarely, variants in *MCEE* (MIM: 608419) result in a milder form of iMMA [3,8,9]. The *MMUT*, *MMAA*, and *MMAB* genes account for approximately 60%, 25%, and 12% of iMMA cases, respectively [1].

The *MMUT* gene (NM_000255.4), located on chromosome 6p12.3, contains 13 exons (exon 1 non-coding) and encodes a 750–amino acid enzyme with an N-terminal substrate-binding domain (residues 1–481) and a C-terminal cofactor-binding domain (residues 586–750), linked by residues 482–585 [10]. The *MMAA* gene (NM_172250.3) on 4q31.21 comprises 7 exons, and the *MMAB* gene (NM_052845.4) on 12q24.11 contains 9 exons encoding a 250–amino acid protein. Molecular genetic confirmation is essential for accurate subtype classification, guiding genetic counseling and facilitating prenatal diagnosis.

Despite growing global knowledge, molecular data from Southeast Asia remain scarce. To address this gap, we report the first detailed clinical and genetic characterization of iMMA in Malaysian children, including two novel *MMUT* variants, and highlight implications for diagnosis and management.

## 2. Methodology

### 2.1. Study Design and Population

This retrospective study was conducted at the Institute for Medical Research (IMR), Kuala Lumpur, as the primary site, with Hospital Tunku Azizah and Hospital Sibu serving as secondary sites. Patients with suspected isolated methylmalonic acidemia (iMMA), referred from hospitals across Malaysia between January 2020 and December 2024, were included if molecular testing confirmed the diagnosis. Clinical suspicion was based on characteristic presentations and biochemical findings such as elevated urinary methylmalonic acid and/or increased blood propionylcarnitine (C3). Confirmation of diagnosis was achieved through molecular genetic testing using a targeted gene panel for MMA-related genes. The study was conducted in accordance with the Declaration of Helsinki and approved by the National Institutes of Health Ethics Committee and Review Board (approval code: 25-01964-C1M (1), approval date: 7 July 2025).

### 2.2. Laboratory Assessment

#### 2.2.1. Biochemical Analysis

Dried blood spot acylcarnitine analysis offers a rapid and sensitive screening approach for numerous organic acidurias and fatty acid oxidation disorders. Liquid chromatography–tandem mass spectrometry (LC–MS/MS) was employed to measure blood amino acids, free carnitine, and acylcarnitines. The reference levels of propionylcarnitine (C3) and C3/acetylcarnitine (C2) ratio in the dried blood spot were 0.22–2.4 umol/L and, 0.02–0.30 respectively. Urinary organic acid analysis using gas chromatography–mass spectrometry (GC-MS), focused on the detection of characteristic metabolites, methylmalonic acid and methylcitric acid. In patients with suspected cobalamin-related defects, plasma total homocysteine and serum vitamin B12 levels were measured to differentiate isolated methylmalonic acidemia (iMMA) from combined MMA with homocystinuria.

To further characterize cobalamin-related disease identified through the above biochemical investigations, vitamin B12 responsiveness was assessed using a standardized protocol. Patients received daily intramuscular hydroxocobalamin (1 mg) for five consecutive days. Urinary organic acids and plasma total homocysteine were measured at baseline and on days 1, 3, 5, 7, and 14. A biochemical response was defined as a >50% reduction in methylmalonic acid and associated metabolites (e.g., 3-hydroxypropionic acid, 2-methylcitrate), accompanied by a decrease in plasma total homocysteine levels.

#### 2.2.2. Molecular Analysis

Genomic DNA was extracted from EDTA-blood using magnetic bead-based kits either Maxwell RSC (Promega Corporation, Madison, WI, USA) or chemagic Prepito (Revvity chemagen Technologie GmbH, Baesweiler, Germany), following standard protocols. A sequential gene testing approach was used, starting with *MMUT*, followed by *MMAA*, *MMAB*, *MMADHC*, or *MCEE*. PCR amplification targeting coding exons and exon–intron boundaries were performed, followed by Sanger sequencing (ABI 3500; Applied Biosystems) and analysis with SeqScape v3 to identify any potential variants. Only *MMUT* and *MMAB* variants were identified and are presented in this report.

Variant interpretation utilized the Human Gene Mutation Database (HGMD; Public version 2023), ClinVar, and literature review, with pathogenicity assessed via Franklin in accordance with ACMG guidelines. Population allele frequencies were referenced from gnomAD v4.1.0, and nomenclature followed Human Genome Variation Society (HGVS) recommendations.

Based on reported family history, targeted familial variant testing by Sanger sequencing was performed for Cases 2 and 7. In Case 2, the patient was screened for the reported familial *MMUT* variants (c.982C>T and c.1106G>A) identified in the affected sibling. In Case 7, the deceased affected sibling had only a biochemical diagnosis of MMA. Both parents were first tested and confirmed as carriers of the reported familial *MMAB* variant (c.644+1G>A), and the patient was subsequently screened for the same variant after birth.

#### 2.2.3. Protein Structural Modeling

Structural modeling was conducted to assess the impact of identified novel variants on the 3D structure of the human methylmalonyl-CoA mutase (MMUT) protein. The crystal structure of the wild-type MMUT protein was obtained from the Protein Data Bank (PDB ID: 2XIQ) [10] and used as a template for homology modeling. Template-target pairwise sequence identity was 94% calculated across the aligned region using Clustal Omega version 1.2.4 [11].

Mutant models, including both deletion–insertion (delins) and missense variants, were generated by first creating modified FASTA sequences corresponding to each variant. These sequences were submitted to the SWISS-MODEL automated homology modeling server (https://swissmodel.expasy.org/, accessed on 1 September 2025) to construct the corresponding mutant protein structures. The energy-minimized wild type and mutant structures were validated using ProCheck [12] and ERRAT [13], accessed via the SAVES v6.0 web server (https://saves.mbi.ucla.edu/, accessed on 2 September 2025). In addition, the APBS (Adaptive Poisson–Boltzmann Solver) Electrostatics Plugin integrated into PYMOL (version 3.1.6.1) (Schrodinger and Delano, 2020) was applied using the pdb2pqr method [14] and default parameters to calculate the electrostatic surface of wild type and mutant MMUT. Electrostatic potentials were mapped onto the solvent-accessible surface using a −5 to +5 kT/e color scale (red = negative, blue = positive). The static models were then analyzed by comparing wild type and mutant structures. Visualization and structural analyses were carried out using PyMOL.

### 2.3. Data Organization

Clinical and biochemical data were obtained from the Biochemistry Unit, Institute for Medical Research (IMR), Hospital Tunku Azizah, and Hospital Sibu, while molecular genetic information was derived from the Molecular Diagnostics Unit, IMR. Data sources included referral forms, biochemical test reports, and genetic analysis records. Each patient’s information was anonymized and compiled into a structured dataset covering key variables: age at diagnosis, sex, clinical presentation, biochemical parameters (C3, C3/C2 ratio, urinary organic acids, plasma total homocysteine, vitamin B12), treatment received, and clinical outcomes. Molecular findings, including identified variants and zygosity, were categorized according to the affected gene and variant type.

### 2.4. Statistical Analysis

This study was descriptive in nature, and no inferential testing were performed. Given the rarity of the condition and the small, clinically heterogeneous cohort, analyses were limited to a descriptive approach to avoid underpowered statistical testing. Clinical, biochemical, and molecular findings are presented as individual case observations. Genotype–phenotype relationships were evaluated qualitatively through comparison of molecular subtypes with age at onset, biochemical vitamin B12 responsiveness, and clinical outcomes. A formal genotype–phenotype correlation analysis was not performed because the study consisted of a small, clinically heterogeneous case series, making statistical testing underpowered and unreliable.

## 3. Results

A total of seven patients were included in this study. All patients were born to healthy and non-consanguineous parents. The clinical history and molecular findings in iMMA patients are summarized in Table 1.

### 3.1. Demographic and Clinical Characteristics

Of the seven patients, five were Iban, one was Malay, and one was Thai-Malay, highlighting a potential population-specific genetic predisposition to isolated methylmalonic acidemia (iMMA) in this region. Among them, three patients were identified as belonging to the Iban ethnic group while the ethnicity of the remaining two was not documented. The age at presentation varied, with the majority (five out of seven) presenting during the neonatal period (Cases 2, 3, 4, 6, and 7), reflecting the classical early-onset form of MMA that typically manifests within the first few days to weeks of life. The remaining two cases (Cases 1 and 5) were diagnosed after the neonatal period, between two months and six years of age. Notably, Cases 2 and 7 were identified through postnatal familial variant testing, prompted by a positive family history of MMA, emphasizing the importance of cascade testing in families with known pathogenic variants.

Early-onset MMA was confirmed in five patients (Cases 3, 4, 5, 6, and 7), most of whom presented within the first four months of life, often with metabolic decompensation. These episodes were frequently triggered by infection, fasting, or poor feeding, and manifested with severe clinical features including hyperammonemia (5/7 patients), metabolic acidosis (4/7 patients), and encephalopathy (4/7 patients). These findings are consistent with the life-threatening metabolic crises that characterize early-onset MMA. Three patients presented with sepsis-like illness, a common mimicker that can delay diagnosis if MMA is not considered in the differential. Other symptoms included recurrent vomiting, poor feeding, altered mental status, seizures, hypotension, pancytopenia, and coagulopathy—highlighting the multisystemic impact of the disorder. Only one patient (Case 1) was classified as late-onset MMA, diagnosed at six years of age with a relatively milder course and better clinical stability.

### 3.2. Biochemical Findings

All seven patients demonstrated elevated levels of propionylcarnitine (C3), along with an increased C3/acetylcarnitine (C2) ratio above 0.2 at initial diagnosis on acylcarnitine profiling performed using tandem mass spectrometry (MS/MS) (Table 2). These findings are in line with impaired propionate metabolism, which occurs due to defective conversion of methylmalonyl-CoA in methylmalonic acidemia (MMA). The uniform elevation of both C3 and C3/C2 across the cohort highlights their diagnostic utility as sensitive and reliable biomarkers for MMA. In clinical practice, these markers serve as a critical first-line screening tool, especially in acutely ill neonates and infants with unexplained metabolic decompensation or neurological symptoms.

Plasma total homocysteine (tHcy) levels were within the normal reference range in all patients for whom data were available, consistent with the diagnosis of isolated MMA (iMMA), which typically presents without homocysteine elevation. tHcy measurements were unavailable for Cases 5 and 7 because the test was not requested by the referring clinicians at the time of initial evaluation, and these values were therefore excluded from descriptive summaries. This finding helped to differentiate iMMA from combined MMA with homocystinuria, such as cblC, cblD, or cblF defects, where elevated homocysteine is a key feature. In two patients, homocysteine data were unavailable due to technical or logistical constraints at the time of diagnosis; however, the remaining patients’ normal tHcy levels strengthened the clinical suspicion of iMMA. Collectively, these biochemical results not only guided appropriate clinical management but also laid the groundwork for targeted genetic testing to confirm the underlying molecular etiology.

### 3.3. Molecular Variant Analysis

**Figure 1:**
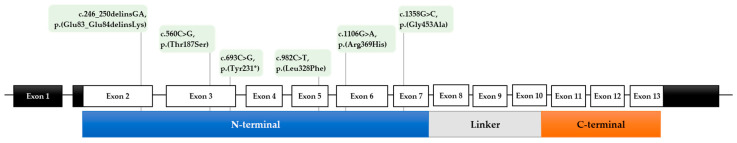
Schematic representation of the variant distribution on the *MMUT* gene (NM_000255.4) identified in this study, mapped onto the N-terminal substrate-binding domain (residues 1–481), linker region (residues 482–585), and C-terminal cofactor-binding domain (residues 586–750) of the methylmalonyl-CoA mutase (MCM) enzyme. The figure is schematic and not drawn to scale. Exons, introns, and untranslated regions are indicated by boxes, lines, and black-shaded areas, respectively.

A total of seven variants were identified through molecular analysis (Table 1), comprising six in the *MMUT* gene and one in *MMAB*. All *MMUT* variants identified in this study were located within the N-terminal substrate-binding domain of the methylmalonyl-CoA mutase (MCM) enzyme (Figure 1).

**Figure 2:**
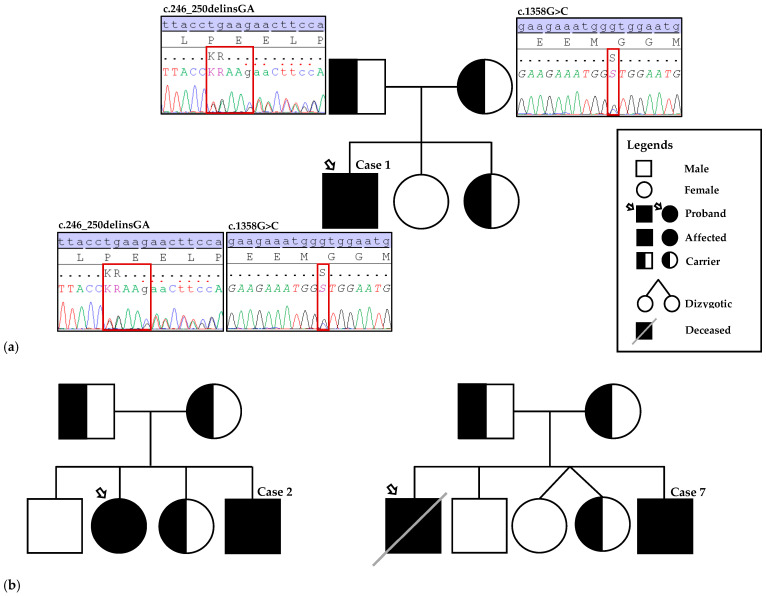
(**a**) Pedigree of the family and Sanger sequencing electropherogram showing the novel *MMUT* variants (c.246_250delinsGA and c.1358G>C) identified in Case 1 (proband). The compound heterozygous variants were inherited from his father and mother, respectively. (**b**) Pedigrees for Cases 2 and 7 illustrating family variant testing based on a positive family history of MMA. Case 2 underwent targeted testing following confirmation of an affected sibling (proband) with the familial *MMUT* variants, c.982C>T and c.1106G>A. In Case 7, the deceased sibling (proband) had only a biochemical diagnosis of MMA without molecular confirmation; both parents were subsequently tested and confirmed to be carriers of the familial *MMAB* variant c.644+1G>A. The paternal great-grandfather and maternal great-grandmother were paternal half-siblings (sharing the same father), representing distant shared ancestry; this relationship is not depicted in the pedigree.

Two variants in *MMUT*, c.246_250delinsGA and c.1358G>C, had not been reported in ClinVar, the HGMD^®^ Public database, or the literature, and were therefore considered novel (Figure 2a). According to ACMG guidelines, the frameshift (indel) variant c.246_250delinsGA is predicted to cause in-frame deletions/insertions in a non-repeat region (PM4), is non-truncating in a mutational hotspot or critical functional domain (PM1), and absent from population databases (PM2), classified as a variant of uncertain significance (VUS) skewed towards likely pathogenic by Franklin. The c.1358G>C, p.(Arg453Pro) variant is a novel missense change in a gene which missense mutations are a common disease mechanism (PP2), predicted deleterious by multiple computational tools (PP3) and absent from population databases (PM2), classified as likely pathogenic by Franklin. The remaining five were published variants, including three missenses (*MMUT*: c.560C>G, c.982C>T, c.1106G>A), one nonsense (*MMUT*: c.693C>G), and one splice-site variant (*MMAB*: c.644+1G>A).

*MMUT*-related iMMA was confirmed in five patients (Cases 1, 2, 3, 4, and 6), while two patients (Cases 5 and 7) harbored *MMAB* variants, consistent with the cblB subtype. Four patients were homozygous for the identified variants, and three were compound heterozygous. Parental testing confirmed compound heterozygosity in two cases (Cases 1 and 2), while for Case 3, parental samples were not available. Pedigrees for Cases 2 and 7 illustrating family variant testing based on a positive family history of MMA are shown in Figure 2b.

### 3.4. Structural Prediction Analysis

#### 3.4.1. Validation of Wild-Type and Mutant Models

The structural quality of both wild-type and mutant MMUT protein models was evaluated and found to be within acceptable parameters, indicating reliable model construction (Table 1). Structural validation was first performed using Ramachandran plot analysis via the PROCHECK tool, which assesses the stereochemical quality of protein structures. In general, the Ramachandran plot has four quadrants which can be used to visualize the allowed and disallowed regions between φ and ψ torsional angles [15]. The Ramachandran plot shows that over 90% of the residues in the wild type and mutant MMUT structures fall within the allowed region, indicating the structure is high quality (Table 3).

Further validation was performed using ERRAT, which evaluates the statistics of non-bonded atomic interactions. ERRAT scores above 50% are considered acceptable, while scores exceeding 95% are indicative of high-quality models [16,17]. The analysis revealed that both the wild-type and mutant models yielded ERRAT scores of approximately 80%, confirming that all structures fall within the acceptable range and in good quality models (Table 3).

#### 3.4.2. Structure Analysis

**Figure 3:**
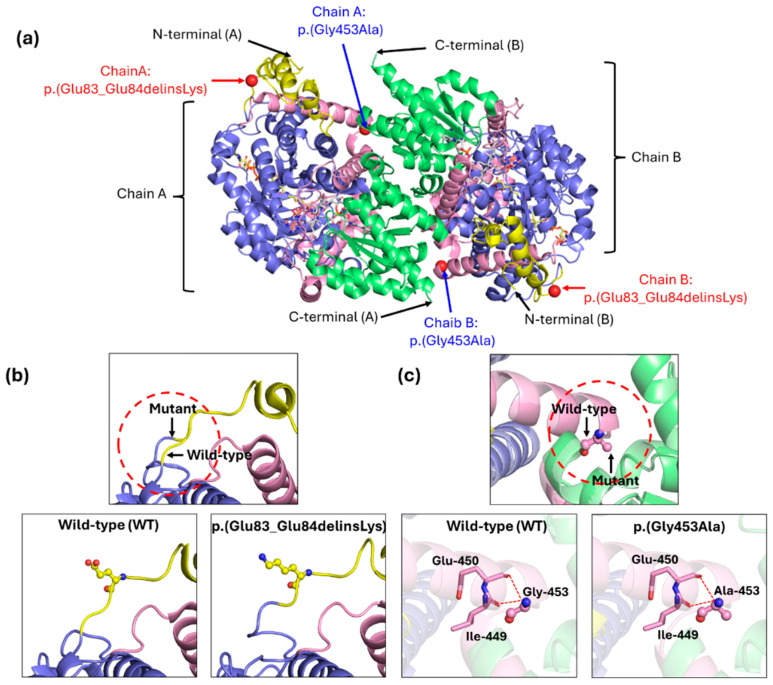
The homodimer X-ray crystal structure of human methylmalonyl-CoA mutase (*MMUT*). (**a**) Localization of variants mapped onto chain A and B of homodimer wild type *MMUT*. The p.(Glu83_Glu84delinsLys) and p.(Gly453Ala) are located at dimerization (yellow color) and linker region (pink color), respectively. (**b**) Superimposition of the deletion–insertion variant, p.(Glu83_Glu84delinsLys) with the wild type shows the shorten of mutant *MMUT* protein structure. (**c**) Superimposition of the missense variant, p.(Gly453Ala) with the wild type introduces a bulkier methyl group in mutant *MMUT* protein. Yellow represents the dimerization region (amino acids: 38–85), blue indicates the catalytic domain (amino acids: 86–423), pink color represents linker region (amino acids: 424–577) and green shows the adenosylcobalamin (AdoCbl) binding domain (amino acids: 578–750). N represents as N terminus and C shows as C terminus. The red dashed lines show the variant changes.

The structural and conformational consequences of the novel variants (delins and missense) in the MMUT protein was analyzed through comparative modeling (Figure 3a). The comparison of the 3D model of the wild-type MMUT protein with its mutants reveals significant changes in molecular interactions among amino acid residues, leading to altered protein conformations.

Electrostatic surface potentials analysis was performed using APBS-plugin PyMOL. In wild-type MMUT protein, residues Glu83 and Glu84 form a small acidic patch that contributes to the local negative (red) electrostatic potential on the protein surface (Figure S1a). In contrast, the p.(Glu83_Glu84delinsLys) variant, resulting from the deletion of the two acidic residues and insertion of a positively charged lysine produces a dramatic local charge reversal (Figure S1b). The acidic patch observed in the wild type is completely abolished and replaced by a strong positive potential (blue) in the mutant surface structure. The deletion of two glutamic acid residues (83 and 84) and replacing with a single lysine alters the N-terminal region of the MMUT (Figure 3b). These residues are located near to the N-terminal domain, which is part of the TIM barrel (α/β barrel) scaffold (Figure 3a). This region contributes to proper folding and stability of the homodimeric-interface structure [10,18]. The loss of two negatively charged amino acids combined with the gain of a positively charged lysine significantly perturbs the local electrostatic environment. Such an electrostatic imbalance may disrupt stabilizing interactions including salt bridges and hydrogen bonds, that are essential for maintaining the structural integrity of the TIM-barrel domain and the dimer interface.

In contrast, electrostatic analysis of the wild-type residue Gly453 and the p.(Gly453Ala) variant shows are nearly identical, with the mutant exhibits minimal changes in surface electrostatic potential (Figure S1c). The missense variant c.1358G>C, p.(Gly453Ala) is located within a critical linker region of the MMUT protein [10,18]. which plays a key role in maintaining conformational flexibility and facilitating interdomain communication (Figure 3a). Glycine at position 453, due to its small size and lack of a side chain, allows for a wide range of torsional angles, enabling the linker to accommodate the dynamic movements necessary for proper domain alignment. Replacement with alanine introduces a methyl group that may increase steric hindrance and reduce conformational flexibility (Figure 3c). This alteration is predicted to disturb interdomain orientation, weaken structural stability, and potentially impair the correct alignment of catalytic elements required for enzymatic function.

## 4. Discussion

### 4.1. Summary of Key Findings

This study aimed to characterize the clinical, biochemical, and genetic features of iMMA in Malaysian children, and to identify novel pathogenic variants. We found two previously unreported *MMUT* variants and a predominance of severe early-onset disease, particularly among Indigenous Sarawakian patients.

The predominance of neonatal-onset presentations underscores the need for heightened suspicion during neonatal metabolic crises. Our findings confirm that elevated C3 and C3/C2 ratios remain reliable screening markers. Definitive diagnosis relies on molecular genetic confirmation, which distinguishes *MMUT*-related disease from cblB defects. These subtypes differ in their prognostic profiles and therapeutic responsiveness, with direct implications for patient management and outcome prediction. Although enzymatic subtyping (*mut*^0^ vs. *mut*^−^) was not available in our setting, subtype interpretation in this cohort was inferred from molecular findings together with biochemical responsiveness.

The overrepresentation of Iban patients in this cohort may reflect referral patterns and geographical factors rather than an inherent ethnic predisposition. The presence of homozygous variants is best explained by shared ancestry and underlying population structure, even in the absence of reported consanguinity. Although no confirmed founder variants for MMA have been documented in the Iban population, population genetic studies indicate that such a possibility is biologically plausible. Notably, Y-chromosomal analyses of Iban, Bidayuh, and Melanau groups in Sarawak reveal uncommon and reduced-diversity Y-STR haplotypes, reflecting demographic structuring that could facilitate the emergence of founder effects [19]. Likewise, broader genomic data indicate that the Iban possess distinctive ancestry profiles and internal population structure when compared with major Peninsular Malaysian groups—namely Malays, Chinese, and Indians—each of which has its own distinct demographic history shaped by different migration waves and admixture patterns [20]. These findings do not confirm a founder effect for the variants observed in our cohort, but they provide valuable context for interpreting the recurrence of identical homozygous variants within this community. To gain a comprehensive understanding of ethnic and regional distribution, assess long-term outcomes and treatment responsiveness, and to confirm the presence of a founder effect, future multicenter, population-based studies incorporating haplotype analysis in larger, ethnically diverse cohorts are essential. Expanding access to molecular diagnostics and implementing robust newborn screening programs hold significant promise for substantially improving the early detection and management of MMA in Malaysia.

Biochemically, all seven patients demonstrated markedly elevated propionylcarnitine (C3) and increased C3/C2 ratios, with urinary organic acids confirming elevated methylmalonic and methylcitric acids. Plasma homocysteine was normal in five patients (Cases 1, 2, 3, 4, and 6), consistent with iMMA and excluding combined forms with homocystinuria, though results were unavailable for two cases (5 and 7).

Identification of two novel *MMUT* variants identified further expands the mutational spectrum of iMMA. Structural modeling supports their pathogenic potential, consistent with the severe phenotypes observed. This highlights the importance of including Malaysian variants in global databases, as underrepresentation of Southeast Asian populations hampers accurate variant interpretation.

These findings underscore the diagnostic value of acylcarnitine and urine organic acid profiling, particularly in settings lacking newborn screening. Furthermore, molecular confirmation is essential for precise subtype classification and timely intervention. Overall, this study highlights the critical role of integrated biochemical and genetic diagnostics in the early detection and personalized management of MMA in Malaysia.

### 4.2. Clinical, Biochemical, and Molecular Spectrum of iMMA in Our Cohort

The clinical and biochemical features in our cohort are consistent with previous reports on methylmalonic acidemia (MMA). Most cases showed the early-onset phenotype, typically linked to severe outcomes such as metabolic crises with hyperammonemia, acidosis, encephalopathy, and sepsis-like presentations [21]. Biochemically, all patients had elevated propionylcarnitine (C3) and increased C3/C2 ratios, confirming disrupted propionate metabolism and supporting the diagnostic role of acylcarnitine analysis [22]. Plasma total homocysteine was normal in most cases, consistent with isolated MMA (iMMA) rather than combined MMA, in line with earlier studies [1].

Despite the small sample size, the *MMUT* variants identified in this study demonstrated mutational heterogeneity. Among the six patients with *MMUT*-related iMMA, four carried missense variants, one had a nonsense variant, and one carried a frameshift (indel) variant. This pattern reflects the known allelic diversity of *MMUT*, with missense variants being the most frequently reported in affected individuals [23]. All identified *MMUT* variants were clustered exclusively within the N-terminal substrate-binding domain (residues 1–481) of the methylmalonyl-CoA mutase (MCM) enzyme (Figure 1), consistent with previous findings that *mut*^0^-associated missense mutations predominantly localize to this region, potentially interfering with substrate binding or leading to protein instability. Notably, no variants were found in the C-terminal cofactor-binding domain or linker region.

The novel *MMUT* missense variant c.1358G>C were found in compound heterozygosity with c.246_250delinsGA in Case 1 (the only late-onset patient). The missense variant c.1358G>C, which results in the substitution of glycine with alanine at codon 453, while the frameshift (indel) variant c.246_250delinsGA is located in exon 2, a known *MMUT* mutational hotspot [24]. To the best of our knowledge, neither of these variants were found in the gnomAD population database, suggesting their rarity. The known *MMUT* variants identified included three missenses (c.560C>G, c.982C>T, c.1106G>A) and one nonsense (c.693C>G). The c.982C>T variant was identified in three patients: homozygous in Cases 4 and 6, and in compound heterozygosity with c.1106G>A in Case 2. Both c.982C>T and c.1106G>A have been associated with the *mut*^0^ enzymatic subtype [23]. The c.1106G>A variant, commonly reported in individuals of Caucasian origin and linked to neonatal onset, further supports its role in severe *mut*^0^ phenotypes [25,26]. The other two known pathogenic variants, c.560C>G and c.693C>G, were detected in a heterozygous state in Case 3. These variants were classified as *mut*^0^ based on enzymatic activity assays [23]. Segregation analysis using parental samples confirmed the inheritance pattern of the identified variants in Cases 1 and 2.

The homozygous c.644+1G>A splice-site variant in intron 8 of the *MMAB* gene was identified in both Iban patients (Cases 5 and 7). This variant was not previously reported in the HGMD, but a single ClinVar submission (VCV000653870.6) notes that it is predicted to disrupt the C-terminal region of the MMAB protein (PMID: 21604717). The pathogenicity of this splice-site change is supported by its expected disruption of the C-terminus, a region essential for proper coenzyme B_12_ processing. Although it is not predicted to undergo nonsense-mediated decay, alteration of this donor site is likely to impair RNA splicing and result in an abnormal MMAB protein. The variant has not been previously reported in individuals with MMAB-related disease and is currently classified as Likely Pathogenic based on limited but consistent evidence. Functional work by Lofgren and Banerjee (2011) [27] demonstrated that C-terminal truncation prevents MMAB from sequestering and delivering coenzyme B_12_ to methylmalonyl-CoA mutase, thereby reducing holo-enzyme formation— a mechanism consistent with the severe biochemical profile observed in Case 5. Although no functional assays were performed for this specific variant, in silico predictions strongly support splicing disruption [28]. Consistent with this, the c.644+1G>A variant showed a high SpliceAI score (0.99), indicating a strong probability of splice-altering effects.

Across the cohort, outcomes varied according to age of onset and molecular subtype. Case 1, the only late-onset patient with biallelic *MMUT* variants, showed no biochemical response to parenteral hydroxocobalamin but has remained clinically stable under conventional metabolic management. He is alive at 11 years of age with mild learning disability and infrequent metabolic decompensations. The favorable outcome in this cobalamin-non-responsive case likely reflects improvements in acute metabolic care, rapid diagnostic work-up, and increased clinician awareness of inborn errors of metabolism [22]. Among the early-onset cases, two patients (Cases 2 and 6) remain alive and clinically stable with no reported neurodevelopmental impairments. Consistent with previous observations, early-onset *mut*^0^ and cblB subtypes showed minimal or absent biochemical responsiveness to hydroxocobalamin. In Case 5, the cblB-deficient patient, carrying homozygous *MMAB* variant c.644+1G>A demonstrated complete non-responsiveness to B12, which aligned with the severe clinical course and fatal outcome [23]. Three patients (Cases 3, 4, and 5) experienced severe neonatal or infantile metabolic crises resulting in early death, while one patient (Case 7) was lost to follow-up. Overall, severe early-onset disease was associated with poor survival, whereas the single late-onset case demonstrated comparatively better neurodevelopmental and clinical outcomes.

### 4.3. Novelty and Added Value of This Study

The predominance of early onset iMMA in our cohort highlights the need for early biochemical and molecular testing in neonates with unexplained metabolic crises. In Malaysia, where newborn screening is not universal, acylcarnitine profiling remains a crucial first-line test, but definitive diagnosis depends on molecular confirmation. Importantly, cascade testing in affected families proved valuable in identifying at-risk individuals via pre symptomatic screening, emphasizing the need to integrate genetic counselling into clinical care pathways.

This is the first genetically confirmed series of iMMA from Malaysia, adding two novel MMUT variants to the international database. By including patients of Indigenous Sarawakian origin, the study contributes to the underrepresented Southeast Asian population in global genomic research. These findings emphasize the importance of expanding variant databases with diverse populations, as reliance on European and East Asian data limits accurate interpretation in other settings.

### 4.4. Strengths and Limitations

#### 4.4.1. Strengths

The main strength of this study lies in being the first genetically confirmed series of iMMA cases reported from Malaysia. By integrating clinical, biochemical, and molecular data, we ensured accurate diagnosis and robust genotype–phenotype correlation. Inclusion of Indigenous Sarawakian patients also expands the representation of Southeast Asian populations in global variant databases. Familial variant testing can help identify at-risk family members early, which is especially important in areas where newborn screening and metabolic care are limited.

#### 4.4.2. Limitations

This study has several limitations. First, the small sample size—while reflective of the rarity of (iMMA)—limits the generalizability of the findings. Although referral bias cannot be fully excluded, its effect is mitigated by the role of the participating tertiary centers as national referral hubs receiving cases from multiple states. Still, some overrepresentation of more severe phenotypes remains possible. We also acknowledge the likelihood of missed cases, including pre-referral deaths and milder or late-onset patients who may never reach tertiary services. Second, the availability of long-term clinical, biochemical, and developmental follow-up data was incomplete for certain patients, particularly those lost to follow-up or diagnosed postmortem. This restricts comprehensive evaluation of the sustained efficacy of treatment regimens and long-term outcomes.

Molecular analysis was limited to five genes, and Sanger sequencing could not detect other variant types such as large deletions or variants in intronic and regulatory regions. The shared homozygous variant observed in some cases is more likely attributable to underlying population structure rather than confirmed parental consanguinity. However, there is still possibility of distant shared ancestry as commonly observed among indigenous groups in Borneo. Enzyme subclassification to confirm *mut*^0^ or *mut*^−^ status was not performed in this study and therefore residual enzymatic activity could not be directly assessed.

Finally, static protein models provide useful initial insights into *MMUT* variants by showing the location of affected residues within the enzyme’s three-dimensional structure and highlighting possible effects on binding pockets, conserved regions, or local interactions. These observations help generate hypotheses about how variants may disrupt folding, cofactor binding, or catalysis. However, such models are limited because they capture only a single conformation and cannot reflect dynamic processes like cofactor movement, loop flexibility, or structural changes during substrate turnover.

### 4.5. Future Directions

Future research should prioritize comprehensive clinical follow-up of iMMA patients to establish the natural history of disease progression in the Malaysian context, including survival, growth, neurological development, and quality of life. Longitudinal biochemical monitoring, particularly of plasma propionylcarnitine (C3), C3/C2 ratio, and urinary organic acids, will be essential to assess treatment response and guide dietary and pharmacological interventions. A recently published protocol for metabolic crisis management is available in the country and is hoped to improve acute care outcomes. Given the predominance of early-onset presentations in this cohort, a universal, expanded newborn screening program using tandem mass spectrometry should be implemented across the country. This would ensure earlier detection and timely intervention, reducing morbidity and mortality in all newborns/neonates in Malaysia. In addition, integration of advanced molecular approaches, including targeted sequencing, whole exome sequencing, and whole-genome sequencing, can improve detection rates and accuracy. Such comprehensive methods are necessary to identify variant types not detectable by Sanger sequencing.

## 5. Conclusions

As the cases were identified through selective screening of symptomatic patients, the observed disease spectrum may not fully represent the true distribution of iMMA in Malaysia. Nonetheless, our findings highlight the importance of strengthening early detection strategies through universal, expanded newborn screening and integrating biochemical and genetic diagnostics into routine clinical care.

## Figures and Tables

**Table 1:** Summary of demographics, clinical characteristics and molecular findings in iMMA Patients.

Case/ Age at Diagnosis	Ethnicity	Family History of MMA	Consanguinity	Gender	Clinical Presentation	Molecular Analysis				Remarks
						Variant	Exon/Intron	Zygosity	Parental Origin	
Case 1/ 6 years	Malay	No	No	Male	Failure to thrive, developmental delay	*MMUT*: c.246_250delinsGA, p.(Glu83_Glu84delinsLys)	Exon 2	Heterozygous	Paternal	Novel variants in this study (VUS skewed towards LP)
						*MMUT*: c.1358G>C, p.(Gly453Ala)	Exon 7	Heterozygous	Maternal	Novel variants in this study (LP)
Case 2/ Day 1	Iban	Yes	No	Male	Pre-symptomatic from high risk screening	*MMUT*: c.982C>T, p.(Leu328Phe)	Exon 5	Heterozygous	Maternal	Reported (ClinVar: VCV000203844.11, HGMD: CM050679)
						*MMUT*: c.1106G>A, p.(Arg369His)	Exon 6	Heterozygous	Paternal	Reported (ClinVar: VCV000203846.47, HGMD: CM990882)
Case 3/ Day 3	Others (Thai-Malay)	No	No	Male	Encephalopathy, seizures, metabolic acidosis, pancytopenia	*MMUT*: c.560C>G, p.(Thr187Ser)	Exon 3	Heterozygous	N/A	Reported (ClinVar: VCV000222914.2, HGMD: CM168550)
						*MMUT*: c.693C>G, p.(Tyr231*)	Exon 3	Heterozygous	N/A	Reported (ClinVar: VCV000222920.5, HGMD: CM164244)
Case 4/ Day 4	Iban	No	No	Female	Sepsis-like illness, encephalopathy, poor feeding, metabolic acidosis	*MMUT*: c.982C>T, p.(Leu328Phe)	Exon 5	Homozygous	Both (Maternal & Paternal)	Reported (ClinVar: VCV000203844.11, HGMD: CM050679)
Case 5/ 4 months	Iban	No	No	Male	Vomiting, hypotonia, encephalopathy, recurrent metabolic decompensation	*MMAB*: c.644+1G>A, p.(?)	Intron 8	Homozygous	Both (Maternal & Paternal)	Reported (ClinVar: VCV000653870.6)
Case 6/ Day 6	Iban	No	No	Female	Sepsis-like features, encephalopathy, metabolic acidosis	*MMUT*: c.982C>T, p.(Leu328Phe)	Exon 5	Homozygous	Both (Maternal & Paternal)	Reported (ClinVar: VCV000203844.11, HGMD: CM050679)
Case 7/ Day 3	Iban	Yes	Distant shared ancestry	Male	Poor feeding, lethargic, tachypnoeic, Specific variant testing performed following family history of MMA	*MMAB*: c.644+1G>A, p.(?)	Intron 8	Homozygous	Both (Maternal & Paternal)	Reported (ClinVar: VCV000653870.6)

Notes: Early-onset cases (diagnosed within the neonatal period) were associated with more severe presentations. Case 1 had the only late-onset presentation and harbored two novel variants. *MMUT* variants were more common overall, while *MMAB* variants were found in two Iban patients, suggesting possible ethnic clustering. Abbreviations: N/A: Not available; VUS: Variant of Uncertain Significance; LP: Likely Pathogenic; p.(?): Predicted protein change is uncertain.

**Table 2:** Summary of Biochemical Profiles and Clinical Outcomes in iMMA Patients.

Case (Age at Diagnosis)	Plasma tHcy Level	Urine Organic Acids	Initial		Post-Treatment		B12 Responsive-ness	Treatment	Clinical Outcome (Age at Last Follow Up)
			C3 (µmol/L)	C3/C2	C3 (µmol/L)	C3/C2			
Case 1 (6 years)	6.0 (Normal)	↑ Methylmalonic acid, ↑ Methylcitric acid	7.88	0.86	0.49	0.02	No	Dietary natural protein restriction (<1.5 g/kg/day); special medical formula restricted in isoleucine, methionine, threonine, and valine; daily carnitine supplement (100 mg/kg/day), daily Carglumic acid (150 mg/kg/day)	Alive, infrequent decompensation, mild learning disability (11 years)
Case 2 (Day 1)	N/A	↑ Methylmalonic acid, ↑ Methylcitric acid	32.00	1.31	18.70	0.29	No	Dietary natural protein restriction (<1.5 g/kg/day), restricted in isoleucine, methionine, threonine, and valine; daily carnitine supplement (100 mg/kg/day), Carglumic acid (100 mg/kg/day)	Alive, infrequent decompensation, normal developmental milestones (5 years)
Case 3 (Day 3)	5.0 (Normal)	↑ Methylmalonic acid, ↑ Methylcitric acid	4.24	0.39	N/A	N/A	No	Acute life support treatment including mechanical ventilation and renal replacement therapy	Died (Day 9) Multi-organ failure
Case 4 (Day 4)	N/A	↑ Methylmalonic acid, ↑ Methylcitric acid	23.73	1.25	N/A	N/A	No	Acute life support treatment including mechanical ventilation and renal replacement therapy	Died (Day 8) Multi-organ failure, sepsis
Case 5 (4 months)	14.0 (Normal)	↑ Methylmalonic acid, ↑ Methylcitric acid	9.40	0.49	22.80	0.74	No	Dietary natural protein restriction (<1.5 g/kg/day); special medical formula restricted in isoleucine, methionine, threonine, and valine; daily carnitine supplement (100 mg/kg/day), daily Carglumic acid (150 mg/kg/day)	Died at 3 years following a severe episode of decompensation resulted in basal ganglia infarct and multi-organ failure
Case 6 (Day 6)	2.0 (Normal)	↑ Methylmalonic acid, ↑ Methylcitric acid	6.84	0.44	N/A	N/A	No	Dietary natural protein restriction (<1.5 g/kg/day), restricted in isoleucine, methionine, threonine, and valine; daily carnitine supplement (100 mg.kg/day), Carglumic acid (100 mg/kg/day)	Alive, infrequent decompensation, mild learning disability (6 years)
Case 7 (Day 3)	12.0 (Normal)	↑ Methylmalonic acid, ↑ Methylcitric acid	11.94	0.75	Defaulted	Defaulted	Not tested *	Lost to follow-up	Lost to follow-up

Abbreviations: Plasma C3: Propionylcarnitine; C2: Acetylcarnitine; Plasma tHcy: Plasma Total Homocysteine; N/A: Not available. Normal range of plasma homocysteine: 5–16 umol/L; Normal range of C3: 0.22–2.4 umol/L; Normal cut-off for C3/C2 ratio: <0.2. * Not tested—lost to follow-up before a trial of therapy could be completed.↑: Increased excretion of methylmalonic acid.

**Table 3:** Quality assessment of the predicted wild type and mutant structures.

Wild Type/Variant	Procheck (Ramachandran Plot Statistic (%))				Errat (%)
	Most favoured	Additionally allowed	Generously allowed	Disallowed	Overall quality factor score
Wild type	91.8	7.7	0.5	0.0	98.1159
p.(Glu83_Glu84delinsLys)	93.0	6.4	0.6	0.0	96.9477
p.(Gly453Ala)	92.1	7.2	0.6	0.0	97.7569

## Data Availability

The data presented in this study are available upon request from the corresponding author due to patient privacy and ethical considerations.

## Supplementary Materials

The following supporting information can be downloaded at: https://www.mdpi.com/article/10.3390/diagnostics16050755/s1, Figure S1: Electrostatic surface potential of wild type and mutants MMUT using the Adaptive Pois-son−Boltzmann Solver (APBS) plugin within PyMOL. (**a**) Wild type, (**b**) p.(Glu83_Glu84delinsLys) variant and (**c**) p.(Gly453Ala) variant. The solvent-accessible electrostatic surface was displayed at range of −5 kT/e (red) to +5 kT/e (blue), indicating the location of negative and positive charges at the protein surface.
