# Supplementary material for: Clinical and Genetic Characterization of Isolated Methylmalonic Acidemia in Malaysian Children: Identification of Two Novel MMUT Variants
Source: Diagnostics (Basel). 2026 Mar 3;16(5):755. doi: 10.3390/diagnostics16050755 (PMC12984186; doi:10.3390/diagnostics16050755)
Supplement: Supplementary file 1 [file diagnostics-16-00755-s001.zip › diagnostics-4002775-supplementary.pdf]

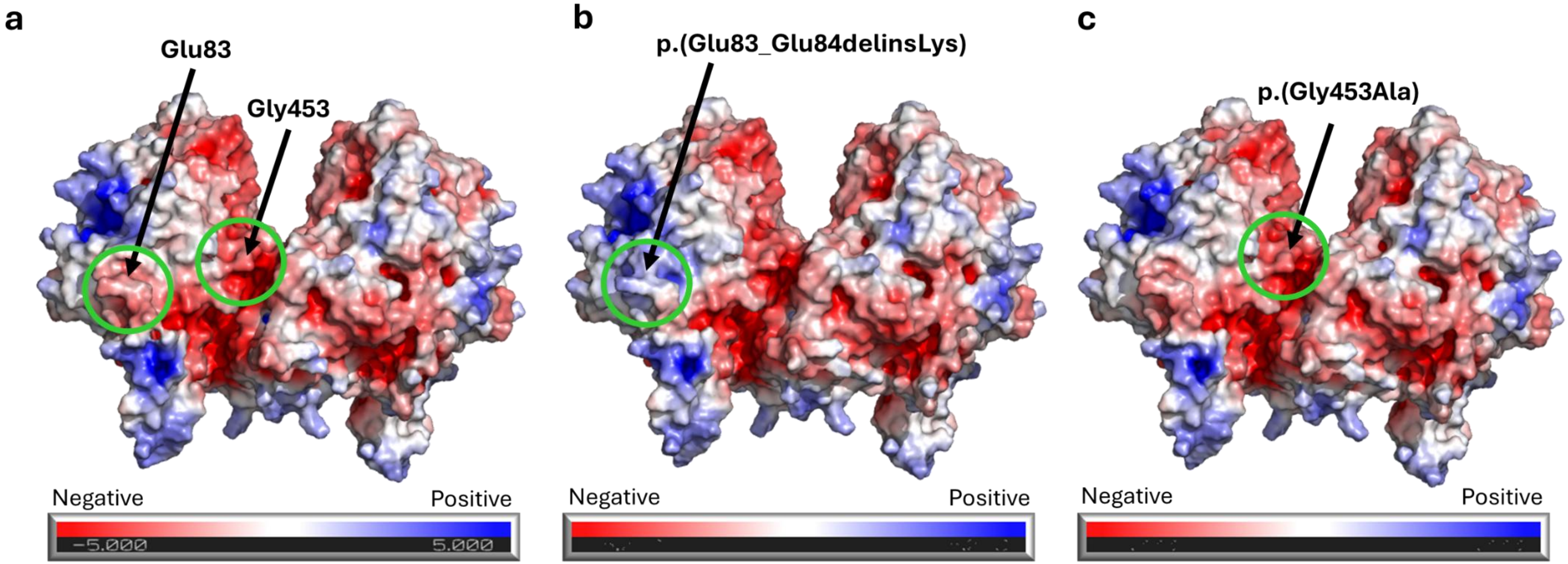

Figure S1. Electrostatic surface potential of wild type and mutants MMUT using the Adaptive Poisson–Boltzmann Solver (APBS) plugin within PyMOL. (a) Wild type, (b) p.(Glu83_Glu84delinsLys) variant and (c) p.(Gly453Ala) variant. The solvent-accessible electrostatic surface was displayed at range of -5 kT/e (red) to +5 kT/e (blue), indicating the location of negative and positive charges at the protein surface.
